# Feasibility of Promoting Smoking Cessation Among Methadone Users Using Multimedia Computer-Assisted Education

**DOI:** 10.2196/jmir.1089

**Published:** 2008-11-03

**Authors:** Joseph Finkelstein, Oleg Lapshin, Eunme Cha

**Affiliations:** ^1^Chronic Disease Informatics ProgramDepartment of General Internal MedicineJohn Hopkins UniversityBaltimoreMDUSA

**Keywords:** Smoking cessation, patient education, computer-assisted instruction, methadone maintenance treatment

## Abstract

**Background:**

The prevalence of smoking is very high among methadone users. As a method of delivering health education, computers can be utilized effectively. However computer-assisted education in methadone users has not been evaluated systematically.

**Objective:**

This study was aimed at assessing feasibility and patient acceptance of an interactive educational module of a multi-component smoking cessation counseling computer program for former illicit drug users treated in an outpatient methadone clinic.

**Methods:**

The computer-mediated education for hazards of smoking utilized in this study was driven by major constructs of adult learning theories. The program interface was tailored to individuals with minimal computer experience and was implemented on a touch screen tablet PC. The number of consecutive methadone-treated current smokers enrolled in the study was 35. After providing socio-demographic and smoking profiles, the patients were asked to use the educational program for 40 minutes. The impact of the computer-mediated education was assessed by administering a pre- and post-intervention Hazards of Smoking Knowledge Survey (HSKS). An attitudinal survey and semi-structured qualitative interview were used after the educational session to assess the opinions of participants about their educational experience.

**Results:**

The computer-mediated education resulted in significant increase of HSKS scores from 60.5 ± 16.3 to 70.4 ± 11.7 with t value 3.69 and *P <* .001. The majority of the patients (78.8%) felt the tablet PC was easy to use, and most of the patients (91.4%) rated the educational experience as good or excellent. After controlling for patient baseline characteristics, the effect of computer-mediated education remained statistically significant.

**Conclusions:**

Computer-assisted education using tablet PCs was feasible, well-accepted, and an effective means of providing hazards of smoking education among methadone users.

## Introduction

Tobacco smoking is one of the major causes of mortality and morbidity among former illicit drug users [1]. The prevalence of smoking among patients on methadone maintenance treatment is very high; nearly 90% of patients who visit methadone clinics smoke tobacco [2]. At the same time only 1 in 3 US methadone maintenance facilities provides smoking cessation counseling for their patients [3]. For the last decade, however, the situation in this field has improved, as more research and organizational efforts have been applied to enhance smoking cessation among methadone users [4-6].

Computers can be a powerful and cost-effective means of providing health education [7]. The necessity for some computer experience, however, was seen as one of the major obstacles to introducing more sophisticated approaches to smoking cessation, such as interactive computer programs and the Internet [8-11]. Methadone-treated patients are frequently from the low-educated, low-income strata of society. Their computer and general reading literacy is often poor, and they usually do not have access to the Internet [12]. To date, the effectiveness of computer-mediated approaches for promoting smoking cessation has not been systematically studied in this population. The potential of interactive, web-based interventions for health education and counseling in this population is unknown. In addition, little systematic information regarding the smoking decision balance of methadone tobacco users is available in the current literature. No data is available about the possible effect of methadone-treated smokers’ socio-demographics, computer literacy, Internet use, and smoking-related behavioral constructs on their ability to successfully use computer-mediated hazards of smoking education. Such information is necessary for developing a targeted anti-smoking, multi-component, computer-mediated program. In this paper we describe development and assessment of an interactive educational module of a multi-component, smoking cessation, counseling computer program for former illicit drug users treated in a methadone outpatient clinic.

The main aims of this study included: (1) development of theoretical framework for computer-mediated hazards of smoking education guided by adult learning theories; (2) implementation of the interactive education program using tablet PC; (3) assessment of the feasibility and patient acceptance of the educational module in methadone-treated patients; (4) collection of systematic information on smoking profiles and attitudes of methadone tobacco users for developing a targeted multi-component, computerized, smoking cessation counseling system; and (5) establishment of possible factors facilitating or impeding successful computer-mediated education in these patients.

## Methods

 Learning theories have been shown to improve significantly the efficacy of educational software [13,14]. Conventional education means, such as lectures, seminars, workshops, books, and videos already incorporate, more or less successfully, practical approaches to learning developed over centuries. Computer-mediated education is an interactive tool, and many approaches that are used intuitively in other spheres may not be applied without a clear understanding and formulation. A computer program has no ability to summarize, repeat, provide feedback or give an additional example if this capacity is not specified and implemented in advance. When designing our learning program, we reviewed over 50 of the most frequently cited theories of adult learning and found that only some of them can be used for constructing a computer program, because many of the theories have no clear experimental support and are applicable only for certain subjects or could be used only under specific conditions or only for a certain part of the learning process. The reviewed theories can be grouped into 4 domains presented in the Table 1.

**Table 1:** Learning theories used in the design of the web-based educational program

Domains	Areas of Concentration	Examples
Cognitive theories	Process of acquisition and organization of knowledge	ACT-R (Adaptive Control of Thought—Rational) Theory [15]; Dual Coding Theory (A. Paivio) [16]
Behavioral theories	Transformation of the outer stimuli into behavior	Connectionism (E. Thorndike) [17]; Contiguity Theory (E. Guthrie) [18]; Drive Reduction Theory (C. Hull) [19]
Humanistic theories	Learning and person, motivation	Experiential Learning (C. Rogers) [20]
Instructional theories	Practical design of learning	Adult Learning Theory (P. Cross) [21]; Conditions of Learning (R. Gagne) [22]

For the purpose of this study, the most important attributes of the existing learning theories were not the general psychological assumptions underlying each theory, but their applicability to the development of interactive educational software. Our analysis of different learning theories resulted in 10 main principles in accordance with which we designed our educational program. These principles are described below.

### Presenting and Explaining the Goals of Learning

Learning is a goal-directed process, as is emphasized in Sign Learning Theory by E. Tolman [23]. The goals of the whole educational program and its parts were explained to the learners on separate screens. The main goal of the program was to increase patient knowledge about the hazards of smoking, and through this, motivate them to quit smoking. This main task was divided into creating sub-goals in accordance with the program structure, into breaking a problem down into subcomponents, and into solving each of those components.

### Clear General Structure of Instruction

The general structure of the educational program should be simple enough to be easily grasped by learners (Constructivist Theory by J. Bruner [24]). Our educational program was structured as a sequence of 6 sections with a final assessment. Each section contained 5 to 9 educational messages with a subsequent multiple-choice question, and had a short 3-4 multiple-choice question quiz at the end.

### Information Chunking

Many theories that exist are concerned with the amount of the information in the educational unit. Chunking of information is the basis for the organization of memory according to the Soar Theory by A. Newell et al [25]. The chunk is a meaningful unit of information, united by meaning, time, location, etc. Ideally, the size of these chunks should be individualized making them suitable for each learner. The Information Processing Theory by G. Miller [26] claims that short-term memory is limited to 7 (or 5 to 9) chunks of information. Therefore, we cut down the educational curriculum into small educational tips, and organized the tips into consecutive sections containing from 5 to 9 tips.

### Enhancing the Cohesion of Knowledge

The presented information for learning should be highly interconnected (Cognitive Flexibility Theory by R. Spiro, P. Feltovitch, and R. Coulson [27]). Therefore, when providing the learner with new information, we referred to facts they had already learned, thus creating links between facts and creating a system of knowledge.

### Case-Based and Problem-Directed Instruction

This approach is very important in order to support learners’ motivation and interest, increase knowledge cohesion, and encourage its transference into the real world. According to such theories as ACT-R (Adaptive Control of Thought—Rational) [28], a theory by J. Anderson, and the previously mentioned Cognitive Flexibility Theory [29], adult learning is better when it is provided in the context of “problem-solving” rather than just being “content-oriented.” Providing short vignettes or more widespread patient cases can also serve this goal.

### Presenting the Most General Ideas First

Following the Subsumption Theory (D. Ausubel) [30], general ideas about smoking were presented first and then specified. At the same time, important concepts were mentioned again in the relevant context with reference to the previously studied material.

### Multiple Representation of Content

According to the Cognitive Flexibility Theory (R. Spiro, P. Feltovitch, and R. Coulson) [31], learning activities should include various representations of content, such as images, audio, and textual information. In our program we used mainly text and images; however, the text was also recorded, and patients had an option to turn the audio on or off. Multiple representation was also important because of the low literacy levels expected in this sample [32].

### Minimizing Working Memory Load

We selected only essential information and presented all information relevant to 1 unit of information in 1 screen. Therefore, we avoided overloading patients with redundant information. Using our program, patients did not need to integrate physically separate sources (for example, combine information presented as series of consecutive hyperlinks or screens). Images and text in our program supplemented each other. All these approaches were used to minimize working memory load, in accordance with Cognitive Load Theory by J. Sweller [33].

### Active Involvement of the Learner in the Learning Process

According to many theories, especially representing behaviorism, learners should actively respond during teaching (Drive Reduction Theory, C. Hull [34]); feedback explaining whether their answers are correct should be provided; and some kind of award or encouragement should be given for correct responses (Operant Conditioning by B. F. Skinner [35]).

### Using Appropriate Socio-Cultural Context

The Triarchic Theory by R. Sternberg [36] requires training to be socioculturally relevant to the learner. The content of the hazards of smoking curriculum was made to address our group of patients who were a predominantly poor, low-educated, African-American, and urban population. The text and images were tailored in accordance to this specific group.

The interactive hazards of smoking education was implemented using the Computer-Assisted Education (CO-ED) system, which has been described in previous studies [14,37]. The CO-ED system provides multimedia, self-paced health education guided by adult learning theories [38,39]. Particular attention has been given to development of a self-explanatory, user-friendly interface oriented towards users with minimal computer skills and limited educational background. The user interface implementation was guided by usability principles for technology designed for individuals with certain limitations in cognitive, perceptual, and motor skills [40]. Overall, the user interface was required to comply with the following principles: (1) Provide equivalent alternatives to auditory and visual content; (2) Don't rely on color alone (provide redundant cues); (3) Provide context and orientation information; (4) Provide clear navigation mechanisms; (5) Ensure that documents are clear and simple; (6) Use large areas of white space and small blocks of text; (7) Provide larger graphics and click targets; (8) Use contrasting foreground and background colors; (9) Minimize blinking images and animation; and (10) Use at least 12-point size fonts and avoid using too many different fonts.

**Figure 1:**
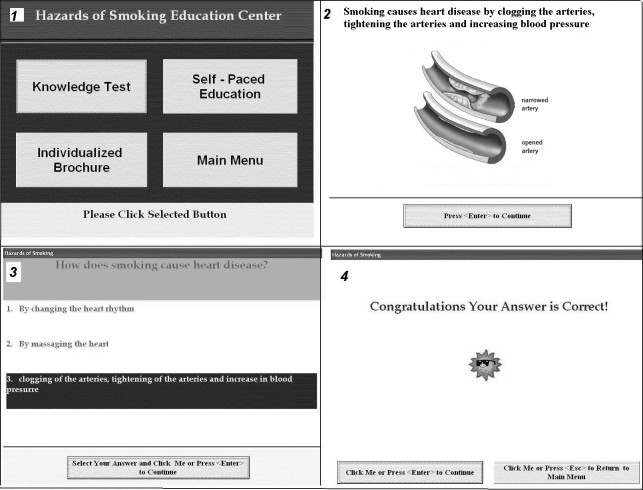
Selected screens of the computer program used in the study: (1) the start screen of the program; (2) educational message; (3) multiple-choice question; and (4) correct answer screen

A touch-screen tablet PC was chosen as a computer platform for this project, based on our previous successful experience in using mobile devices for health education in low-income inner-city populations [39,56]. This platform allowed us to successfully implement software which was compliant with the above mentioned usability principles. The small size of the tablet PC also allowed us to minimize the space necessary for conducting the study. Since touch-screen technology has already been successfully introduced to the general population, the time required for training to use the computer and the educational program was very limited. The low reading literacy of this particular group of patients was addressed by using large fonts (36 pixels and larger), maintaining text readability at a fifth-grade level, and providing audio functionality with all text read aloud. The patients could turn this function on or off depending on their needs. Only 1 educational message was displayed per screen, allowing for both an increase in readability and a decrease in work memory load. Each structural field of the screen was color-coded (ie, fields with educational messages, multiple choice questions, answers, and prompts each had its own color to make it easier to identify them intuitively when moving from screen to screen). Screen navigation was streamlined and tested to make it error proof. Each screen had only 1-2 options leading to another screen, and no combination of actions could possibly lead to an error. Figure 1 shows the appearance of the start screen, educational message screen, multiple-choice question screen, and feedback screen.

**Figure 2:**
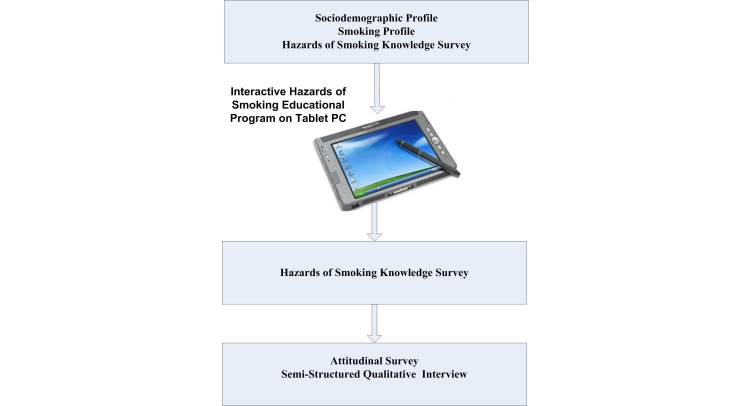
The design of the study

The study utilized quasi-experimental pre/post design [41,42]. From an outpatient methadone maintenance treatment clinic located at downtown Baltimore, 35 consecutive methadone-treated current smokers were recruited. All study protocol was carried out during a single patient visit (Figure 2). At the beginning of the study, sociodemographics and smoking profiles were collected, and a hazards of smoking knowledge survey was administered. Following the baseline interview, the patients were asked to spend 40 minutes on the hazards of smoking education program installed on a tablet PC. Immediately after completion of the educational session, the patients were asked to complete the hazards of smoking knowledge survey again. Finally, an attitudinal survey and semi-structured qualitative interview were administered to assess patient acceptance of the computer-mediated educational program.

The baseline interview included information on patient socio-economic status, demographic, computer literacy, and smoking profile. The smoking profile consisted of smoking history and major behavioral factors known to affect interest in smoking cessation. The behavioral factors were assessed using the Stages of Change Scale, Smoking Self-Efficacy Questionnaire and an open-ended decision balance survey. The hazard of smoking knowledge survey was used to assess the efficacy of the computer-mediated intervention. The baseline variables were utilized to ascertain whether sociodemographic or behavioral factors affect the education program outcomes.

The Hazards of Smoking Knowledge Survey (HSKS) contained questions about hazards of smoking information recommended for patient education by the Department of Health and Human Services and Centers for Disease Control and Prevention guidelines [43,44]. The knowledge survey consisted of 35 basic “true” or “false” statements about smoking and its harmful health effects. The final score of this survey was calculated as a percentage of correct answers. Separate scores were also calculated for 4 major topics of the hazards of smoking educational curriculum based on a corresponding subset of questions from the knowledge survey: (1) general information about tobacco smoking; (2) health consequences of smoking; (3) nicotine addiction; and (4) quitting smoking.

The Stages of Change Scale [45,46] has been widely used to measure readiness to quit smoking according to Prochaska and DiClemente’s Transtheoretical Model [47]. The scale was shown to have good psychometric properties and external validity [48]. Its test-retest reliability (kappa) was 0.78 in a sample of 404 Australian smokers [49].

 The Smoking Self-Efficacy Questionnaire (SEQ-12) measures the confidence of current and former smokers in their ability to abstain from smoking in high-risk situations [50]. Lower self-efficacy means that an individual is more tempted to smoke, and vice versa. The SEQ-12 includes 2 sub-scales which measure ability to refrain from smoking when facing internal or external stimuli. The questionnaire has excellent psychometric properties with internal consistency coefficients above 0.94 [50].

The smoking decision balance was ascertained using a patient-administered survey including open-ended questions. The smoking decision balance [51] plays an important role in smoking decision-making according to the Transtheoretical model that is widely used for smoking cessation [52]. The patients were asked (1) to describe the things about smoking that they like and do not like; (2) to explain what they would like about quitting smoking, and what would worry them or would be difficult about quitting smoking; and (3) to describe barriers, triggers, and possible coping mechanisms related to smoking cessation. The patient answers were coded and analyzed using HyperRESEARCH software. The positive and negative factors affecting the decision to quit smoking were summed up in 2 separate variables characterizing the total number of facilitating and inhibiting factors to quit.

An attitudinal survey and semi-structured qualitative interview were administered after the educational session to assess the opinions of participants about their experience. The attitudinal survey was aimed at grading patients’ acceptance of the computer program, and their perceptions of its usability and user-friendliness. The semi-structured qualitative interview was used to elicit subjects’ perceived limitations and concerns about the hazards of smoking program, and to identify directions for future improvements. The attitudinal survey [14,56] and qualitative interview [53-55] were used successfully in our previous studies to evaluate patient acceptance and attitudes towards new computer technology.

All statistical analyses were performed using SAS software version 9.1. Frequencies and percentages were calculated for all categorical variables. Means, medians, SDs, and ranges were computed for continuous variables. Inferential statistics included analysis of variance (ANOVA), and *t* tests. As a 1-factor experiment, ANOVA was used to test for differences between 2 groups (before and after), controlling for education, age, income level, gender, job status, frequency of computer use at home and at work, frequency of using the Internet and ATM machines, smoking stages of change, and number of facilitating and inhibiting factors of smoking cessation. ANOVA was performed using PROC GLM in SAS for unbalanced designs. Qualitative data were transcribed, coded, and analyzed with qualitative analysis software HyperRESEARCH version 2.5.

## Results

The study sample consisted of 23 women (65.7%) and 12 men, 45.4 ± 6.7 years old, with an average 11.2 ± 1.7 years of education. Most of the patients were unemployed and had income of $20,000 a year or less (Table 2). Most of the patients (21/35, 60.0%) had never used computers in their lives.

The patients had smoked, on average, for 23.2 ± 10.2 years. Those patients who had discussed their smoking at least once with their doctors numbered 20 (20/35, 57.1%), although it was not connected with their knowledge about hazards of smoking or their stages of change. More than half of the patients considered smoking to be a severe problem for them, while only 1 patient thought it was not a problem at all. The total smoking self-efficacy score was 27.3 ± 12.4, which corresponds to a low perceived ability to abstain from smoking. According to Prochaska’s Stages of Change Scale, 8 patients were in the preparation stage (8/35, 22.9%), the majority were in the contemplation stage (16/35, 45.7%), and the remaining patients were in precontemplation (10/35, 28.6%).

The odor of smoke was the most frequently reported unpleasant effect of smoking (12/35, 34.3%), along with breathing and lung problems (Table 3). The main trigger to smoke was feeling nervous or depressed (25/35, 71.4%). About 20% of participants (7/35) expected to become nervous or depressed after quitting. Feeling relaxed or calm was the most frequently reported benefit of smoking (23/35, 65.7%). No desire to quit was the most frequently cited barrier to quitting, reported by 6 patients (6/35, 17.1%) (Table 3)*.*

**Table 2:** Baseline characteristics of the study sample (N=35). See text for continuous variables (age, education, years of smoking).

Categorical Variables	% (N)
**Sex**	
Male	28.6% (10)
Female	65.7% (23)
Missing	5.7% (2)
**Income level**	
No Income	8.6% (3)
<20K	62.8% (22)
20K-30K	5.7% (2)
30K-40K	2.9% (1)
40K-50K	2.9% (1)
Missing	17.1% (6)
**How much do you smoke?**	
½ pack a week	2.9% (1)
½ pack a day	25.7% (9)
1 pack a day	57.1% (20)
2 packs a day or more	14.3% (5)
**Job**	
Permanent	17.1% (6)
Temporary/part-time	5.8% (2)
None	77.1% (27)
**Internet Use**	
Never	74.3% (26)
Once a month or less	11.4% (4)
Once a week	5.7% (2)
Once a day	8.6% (3)
**Computer use**	
Never	60.0% (21)
Once a month or less	20.0% (7)
Once a week	5.7% (2)
Once a day	14.3% (5)
**Self-reported knowledge about quitting smoking**	
None	11.4% (4)
Very limited	62.9% (22)
Good	22.8% (8)
Missing	2.9% (1)
**How severe of a problem do you consider your smoking?**	
Not a problem	2.9% (1)
Mild problem	14.3% (5)
Moderate problem	25.7% (9)
Severe problem	57.1% (20)

**Table 3:** Smoking decision balance (patients could mention as many benefits or negative consequences as they liked)

	Frequency (N = 34)
**Benefits of smoking**	
Relaxation, calming affect	23 (67.6%)
Pleasure	7 (20.6%)
Taste	7 (20.6%)
Helps digest food	4 (11.8%)
Appearance when you are smoking	2 (5.9%)
Feeling refreshed	2 (5.9%)
Process of smoking itself	2 (5.9%)
Being a part of the group	1 (2.9%)
**Benefits of quitting**	
Good for health in general	7 (20.6%)
Decreased breathing problems	7 (20.6%)
Saves money	11 (32.4%)
No odor of smoke	9 (26.5%)
Better taste of food	5 (14.7%)
Other specific health benefits	4 (11.8%)
Better appetite	3 (8.8%)
Psychological benefits	2 (5.9%)
**Barriers to quitting smoking**	
No desire to quit	6 (17.6%)
Expected weight gain	5 (14.7%)
Temptation to smoke	5 (14.7%)
Stressful life	4 (11.8%)
Need help to quit	3 (8.8%)
Want to be a part of the group	3 (8.8%)
Other health consequences of quitting	3 (8.8%)
Other barriers	3 (8.8%)
**Strategies to cope with smoking urges**	
Substitute with meals or chewing gum	17 (50.0%)
Substitute with other activities	13 (38.2%)
Not to be around smokers	7 (20.6%)
Getting help or medications	4 (11.8%)
Other coping behaviors	5 (14.7%)
**Negative consequences of smoking**	
Odor of smoke	12 (35.3%)
Breathing and lung problems	12 (35.3%)
Possibility of cancer	6 (17.6%)
Bad for health in general	6 (17.6%)
Expensive	6 (17.6%)
Bothers other people	5 (14.7%)
Yellow teeth color	5 (14.7%)
Bad breath	5 (14.7%)
Other health problems	3 (8.8%)
Burned clothes	3 (8.8%)
Bad taste of food	2 (5.9%)
Other	3 (8.8%)
**Negative consequences of quitting**	
Nervousness or depression	7 (20.6%)
Weight gain	6 (17.6%)
Urges when smelling odor of smoke	5 (14.7%)
Temptation to smoke	4 (11.8%)
Being among smokers	4 (11.8%)
Other	2 (5.9%)
**Smoking triggers**	
When nervous or depressed	25 (73.5%)
After eating a meal	22 (64.7%)
In the morning or after waking up	21 (61.8%)
When using bathroom	6 (17.6%)
When with friends or other people	5 (14.7%)
At night or before bed	5 (14.7%)
During relaxation or rest	4 (11.8%)
After drinking alcohol	4 (11.8%)
At noon	4 (11.8%)
Other triggers	4 (11.8%)

Although the questions asked were very basic and simple, the average knowledge score at pre-test was 60.5 ± 16.3 points (corresponding to the percentage of correct answers). Participants scored lower than the total average for questions under category of ‘Nicotine addiction,’ and ‘Quitting smoking.’ Examples of quiz statements the validity of which many patients could not assess correctly for ‘Nicotine addiction’ include ‘Nicotine is an addictive chemical’ and ‘Nicotine causes the arteries to loosen and allow more blood flow’; for ‘Quitting smoking’, examples are ‘Quitting smoking starts to benefit your health about 1-2 years after quitting’ and ‘If you smoked for more than 10 years quitting won’t help’. As a result of computer-mediated education, participant knowledge about the hazards of smoking significantly increased to 70.4 ± 11.7 points, *P* < .001. The increase in knowledge was not associated with the patients’ level of education or computer experience. In 3 major curriculum topics out of 4, the increase in knowledge scores was statistically significant (Table 4).

**Table 4:** Hazards of smoking knowledge scores before and after the intervention (N = 34)

Topics	Pre-test Post-test	T value (*P*)
1. General information about tobacco smoking	69.6 ± 16.7 79.9 ± 11.4	2.97 (.004)
2. Health consequences of smoking	58.8 ± 19.1 70.6 ± 14.4	2.87 (.006)
3. Nicotine addiction	41.9 ± 30.0 55.9 ± 23.9	2.13 (.04)
4. Quitting smoking	47.1 ± 28.7 58.1 ± 22.0	1.78 (.08)
Total	60.5 ± 16.3 70.4 ± 11.7	3.69 (< .001)

In order to establish factors facilitating or impeding successful computer-mediated education, analysis of variance (ANOVA) was employed. Considering the study design as a 1-factor experiment, ANOVA was used to test for differences between pre- and post-test knowledge scores controlling for sociodemographics, computer experience, smoking history, stages of change, and number of perceived inhibitors and facilitators of smoking cessation. The education was stratified into ‘less than high school’ and ‘high school or more’ groups. Age was categorized as < 45 and ≥ 45 groups. The income was divided into 3 groups: no income, < 20K, and ≥ 20K. Job status was stratified into ‘employed’ and ‘no job’ and the amount of smoking was categorized as ‘2 packs a day or more’, ‘1 pack a day’, ‘1/2 pack a day’, and ‘1/2 pack a week.’ Frequency of using the Internet or a computer was categorized into 2 groups: ‘never’ and ‘use at least once a month’. The stages of quitting smoking were categorized into 3 groups: pre-contemplation, contemplation, and preparation. Patients were asked to list any factors that inhibit and facilitate their intentions to quit smoking. The number of these factors was introduced into the model, which were stratified into 2 groups using their median value as a dividing point (the number of inhibitors: 1-7 and > 7, the facilitators: 1-6 and > 6). After controlling for all these variables, the difference between pre- and post-test knowledge scores remained significant (*P =* .004). The variables which significantly affected knowledge gain were gender (males were more likely to have a higher knowledge gain than females, *P =* .015), age (people over 45 were less likely to have a knowledge gain, *P =* .02), stage of change (subjects in contemplation were more likely to have higher knowledge gain, *P =* .03), and the number of facilitating factors to quit smoking (patients with more facilitators were more likely to study more successfully, *P =* .002). Patient education level, computer experience, and Internet use did not affect the results of computer-assisted education.

Acceptance of the program interface according to the Attitudinal Survey is presented in Table 5. The program was very well accepted by the overall participants. One patient did not complete the attitudinal survey and another patient missed filling in questions #9 and #18. Therefore, percentages for questions #9 and #18 are calculated based on 33 participants and the rest are calculated based on 34 participants. For 78.8% (26) of patients, using the computer was not complicated at all. Most of the patients (32 persons, 94.0%) rated the program as good or excellent. The majority of patients had little or no problem understanding the presented information, but 27.3% (9 patients) encountered very significant amounts of unknown words. About 18% (6) of patients claimed that they encountered information which was difficult to understand, and 11.8% (4) frequently found it confusing (Table 5).

Patients’ feedback about their learning experience was ascertained using semi-structured qualitative interviews. Except for a short training session (~ 10 minutes) provided by a research assistant, 28 (82.4%) participants did not need any help during the educational session. One participant did not complete the qualitative interview; therefore percentages are calculated based on 34 participants. Only 7 (20.6%) patients felt tired at the end of the educational session, and 5 (14.7%) believed the educational session was too long. Regarding ways to improve the program, 7 (20.6%) patients thought they would prefer to listen to the educational messages; 18 (52.9%) thought they would like to both read and listen to the information; and 27 (79.4%) thought that including more video clips would make the program better. For 31 (91.2%) patients it was amenable to answer a multiple choice question after each educational message; 26 (76.5%) were compliant with the conditional self-quiz design of the educational session (ie, patients had to demonstrate sufficient knowledge of a module before being allowed to move to the next one); and 24 (70.6%) patients felt “it was ok to repeat the whole section again.” Finally, more than half of the patients (20, or 58.8%) preferred using a computer program to learn about smoking over more traditional types of education, such as brochures, videotapes, healthcare providers, and browsing the Internet.

**Table 5:** Patient satisfaction and attitudes toward different aspects of their educational experience according to the attitudinal survey

#	Question		Option^†^ (%)			
		Total N	1	2	3	4
1	How complicated was it to use the computer?	34	11.8	5.9	2.9	79.4
2	Did you have any difficulty in moving from one screen to another?	35	91.3	2.9	2.9	2.9
3	How difficult was it to use the keyboard/mouse?	35	2.9	5.9	2.9	88.3
4	Did you have any difficulties in reading text from the computer screen?	35	91.2	5.9	0.0	2.9
5	Was the size of the text presented on the screen sufficient?	35	85.3	5.9	2.9	5.9
6	Did you like the colors used on the computer screen?	35	85.3	8.8	5.9	0.0
7	Did you like the audiovisual content provided by the computer?	35	91.2	5.9	0.0	2.9
8	Did you get all the necessary information about using the computer during initial practice session?	34	91.2	5.9	0.0	2.9
9	Did you come across any unknown words which were not explained by the computer?	33	27.3	6.1	12.1	54.5
10	How difficult were the sentences used in the educational materials?	34	5.9	5.9	8.8	79.4
11	How much new information did you get using the computer?	35	67.7	23.5	5.9	2.9
12	Did you get any feedback from computer about your learning progress?	35	67.6	14.7	11.8	5.9
13	How frequently did you find the information confusing?	35	11.8	17.6	14.7	55.9
14	How frequently did you find educational contents difficult to understand?	35	17.6	8.8	26.5	47.1
15	Did you have to wait for new information to come up on the screen?	35	11.8	5.8	11.8	70.6
16	Would you like to use this educational program in the future?	35	100.0	0.0	0.0	0.0
17	Would you advise other patients to use this educational program?	34	91.2	8.8	0.0	0.0
18	Overall how would you grade this educational program?	33	3.0	3.0	18.2	75.8

^†^The following options were used for the questions above (in the ascending order): #1: Very complicated, Moderately complicated, Slightly complicated, Not complicated at all #2, #4: Not at all, Very rarely, Frequently, All the time #3, #10: Very difficult, Moderately difficult, Slightly difficult, Not difficult at all #5: Fully sufficient, Sufficient almost all the time, Sufficient some of the time, Not sufficient at all #6, #7: Certainly yes, To a large extent, To some extent, No #8: All information, Almost all information, Partial information, Very limited information #9: Very significant, Considerable, A few, None #11: Very significant amount, Considerable, Little, Very little #12, #15: All the time, Occasionally, Very rarely, Never #13, #14: Very frequently, Occasionally, Very rarely, Never #16, #17: Certainly yes, Maybe, Unlikely, No #18: Needs serious improvement, Satisfactory, Good, Excellent

## Discussion

Computer-assisted patient education utilizing the main concepts of adult learning theories was successfully implemented in methadone-treated smokers. The results showed statistically significant increases in the subject knowledge scores. This result remained statistically significant after adjusting for major sociodemographic factors, smoking profile, and behavioral factors. The majority of the patients were able to navigate successfully the user interface even though most of them had never used computers before. These results corroborate our previous findings in which we showed that even low-income individuals with limited education and no previous computer skills were able to navigate successfully an educational computer program after 5-15 minutes of supervised training, when specific user interface principles were implemented [38,39,56].

Former drug users on maintenance methadone treatment are quite different from other groups of patients who need smoking cessation interventions. They are often depressed and have other psychiatric problems including chemical and non-chemical dependencies. Our research team is in the process of the development of a comprehensive computerized smoking cessation program. Testing of the educational module of this program was done to get feedback from patients about its acceptability and design, and also to elicit additional information about their smoking profiles. After only a brief training session, the patients, most of whom were using computers for the first time in their lives, were able to go through the educational program. Our education intervention was successful, and the program installed on tablet PCs was very well accepted by the patients.

Learning theories cannot serve as a universal recipe or magic pill to improve patient education [57]; rather they should be used and applied thoughtfully and selectively [13]. In designing our educational program we used a mix of ideas derived from different theories. Though this approach prevented us from assessing which particular ideas mostly contributed to outcomes, we thought that for the success of the intervention it was more important to provide a theoretical framework incorporating a broad spectrum of ideas and, therefore, allowing inclusion of a more diverse population. Other studies tested applicability and efficiency of some specific theories to patient education, such as the problem-based approach [58]. Using a combination of approaches can potentially be more effective when target users are represented by diverse clientele in various settings where education is implemented [59-61].

When constructing the program to install on tablet PCs we also took into account the low educational level of the patients in methadone outpatient clinics. Such design details as using self-explanatory error-proof navigation, large fonts, and audio support substantially improved the usability of the program and were highly valued by the patients.

Patients in our sample lacked basic knowledge about hazards of smoking and smoking cessation, and underestimated the negative health effects of smoking. Only 6 participants recollected such long-term consequences of smoking tobacco as lung cancer, and only 1 patient was worried by the possibility of heart problems caused by smoking. This supports the previous data [62] that former drug abusers frequently underestimate the risks of smoking. The odor of smoke was the most frequently reported negative side effect of being a smoker. Only 4 (11.4%) patients thought that getting help from others or taking some kind of medication could aid in coping with smoking urges.

Our data agree with Nahvi et al [6] that methadone users are interested in smoking cessation. In that study, nearly half of the smokers were in the contemplation stage, and about 20% were in the preparation stage, corroborating our results. These results are very interesting, when one considers that these people are already struggling with at least one kind of addiction, have numerous psychiatric comorbidities, are subjected to a lot of stress in their lives, and are frequently unemployed. Our data helps with understanding why it can be so difficult for these patients to decide to quit. Depression and nervousness are seen as major consequences of quitting, while at the same time these feelings are triggers for the desire to smoke.

In this study the main outcome was knowledge gain in hazards of smoking. We did not expect after only a single brief intervention to motivate patients to decide to quit smoking. We see the computer-assisted hazards of smoking education as a component of a comprehensive smoking cessation program, including computerized and/or in-person counseling. Tailoring computer-mediated smoking cessation counseling to a certain stage of change, gender, cultural background, and smoking profile can facilitate smoking cessation.

The content of our educational curriculum was simplified by adjusting the readability of the content to the fifth-grade level. Despite this, according to the attitudinal survey, reading comprehension was one of the major problem areas in this pilot study. Readability and accessibility can be an issue for a variety of consumer health applications, and there are well established approaches on how to measure and avoid this problem [63,64]. We think that this group of patients requires additional measures to facilitate learning (eg, audiovisual aids and significantly simplified grammar)*.* Most of the patients were not used to studying and had no previous computer experience. Despite that, most of them were not tired at the end of the educational session. Our initial concerns that the ‘exam-like’ format of the program may not be the best fit for this group of patients were not supported by our findings. An absolute majority of the patients accepted multiple-choice questions and quizzes very well. When asked, patients preferred computer-assisted education to other conventional means of education.

Different computer-mediated smoking cessation approaches have been shown to be successful. These include computer-generated individually tailored letters [65] and internet-based smoking cessation programs [66-69]. Educational computer programs about the hazards of smoking can be used separately or as a part of smoking cessation computer intervention in outpatient drug treatment facilities. The same model can be applied to other types of health behaviors, such as healthy nutrition, alcohol drinking, condom use, and physical activity, all of which also constitute significant problems for former drug users [70,71]. Internet-enabled, touch-screen computers could be easily utilized for a widespread dissemination of computer-assisted health education in methadone clinics.

When analyzing which factors influence knowledge gain in the sample, we found that age, gender, stage of change, and number of facilitators (potentially beneficial consequences of quitting smoking for patients) influenced it. Neither computer/Internet experience nor the level of education was significant for patient ability to learn. We may conclude that the program was simple and effective enough for the patients independent of their education and computer skills, but patients who perceived smoking cessation more positively demonstrated a higher a knowledge gain. Our results supported the notion that adult learning theories could provide an effective framework for successful computer-mediated education in a group as challenging as methadone-treated smokers.

### Conclusions

Computer-assisted education using tablet PCs was a feasible, well-accepted, and effective means of providing hazards of smoking education among methadone-treated smokers. Simplifying the content of the educational curriculum, utilizing the main concepts of adult learning theories, and using a self-explanatory multimedia user interface can make computer-assisted education more effective in this group of patients.

## Abbreviations

ACT-R Theory: Adaptive Control of Thought—Rational Theory
ANOVA: analysis of variance
CO-ED: computer-assisted education
HSKS: hazards of smoking knowledge survey
SEQ-12: smoking self-efficacy questionnaire
